# Remembering Dr. Ann-Marie Widström

**DOI:** 10.1177/0890334421995127

**Published:** 2021-03-22

**Authors:** Louise Dumas

**Affiliations:** 1 59310 Université du Québec en Outaouais, Gatineau, Quebec, Canada

**Keywords:** breastfeeding, infant development, lactation


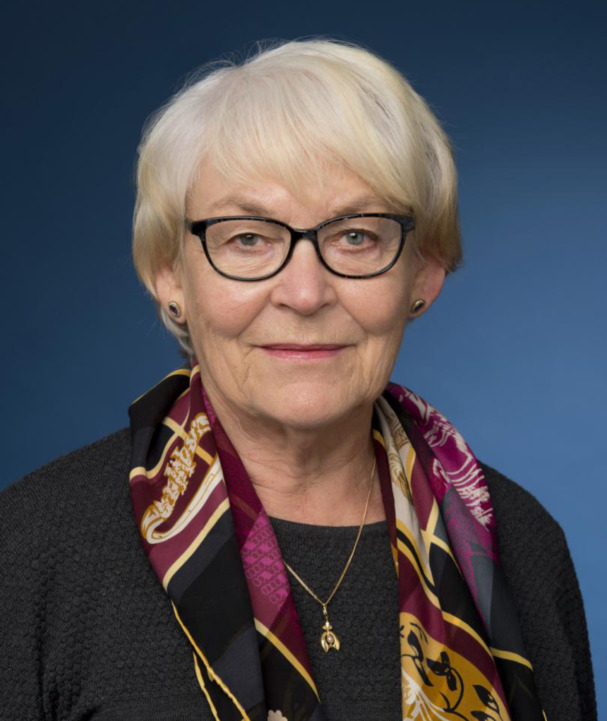


On January 8, 2021, newborns around the world lost one of their greatest advocates: Ann-Marie Widström died at age 85, in the presence of her dearest family. Health care professionals and families alike are deeply saddened by her passing. I had the privilege to have Ann-Marie as an outstanding mentor and a long-time cherished friend. I write this to honor her memory and remind the world of her great legacy.

As a visionary researcher, Ann-Marie Widström observed, described, studied, and validated what is now called “*the innate sequence of the human mammal at birth*” or “*the nine stages of newborn instinctual behaviour*” leading the newborn to self-regulate at birth and find the breast to breastfeed for the first time.

All mammals have their own innate sequence at birth leading to survival, self-regulation, and feeding. Ann-Marie described the human sequence beginning in 1993 with a short video called *Breastfeeding-Baby’s Choice*, which is now a CD under the same title, translated into many languages, and well utilized throughout the world. She then extensively published her findings in many scientific periodicals, each time further defining and explaining the nine stages, and the importance of implementing this sequence at clinical and research levels.

Ann-Marie was a very meticulous and even perfectionist researcher and writer. She taught us well. Many junior and senior researchers from different countries were inspired and moved by her charisma, her simplicity, and her deep humaneness. She was always open to receiving them in Stockholm, at the Karolinska Institutet, for a few hours, days, or even years, enthusiastically sharing her knowledge, and her curiosity about everything related to newborns. Discussions were always animated and with touches of humor.

As a friend, she was extremely present, open, attentive, warm, lively, and funny. She much enjoyed good meals with friends, with lots of discussion, jokes, and sharing of memories. Ann-Marie, rest in peace. The world has become a better place because of who you were, and because of your work. It was an honor and a pleasure to work with you for more than 10 years and to cherish you as our friend.

